# Breaking the Barrier: SARS-CoV-2 Infections in Wild and Companion Animals and Their Implications for Public Health

**DOI:** 10.3390/v16060956

**Published:** 2024-06-13

**Authors:** Zhandos Abay, Sandugash Sadikaliyeva, Ainur Nurpeisova, Kuanysh Jekebekov, Kamshat Shorayeva, Bolat Yespembetov, Sergazy Nurabayev, Aslan Kerimbayev, Berik Khairullin, Hansang Yoo, Lespek Kutumbetov, Markhabat Kassenov, Kunsulu Zakarya

**Affiliations:** 1 Research Institute for Biological Safety Problems, Guardeyskiy uts 080409, Kazakhstan; 2 MVA Group Scientific-Research Production Center Ltd., Almaty 050046, Kazakhstan; 3 College of Veterinary Medicine, Seoul National University, Seoul 08826, Republic of Korea

**Keywords:** SARS-CoV-2, One Health, companion animals, transmission, risk

## Abstract

The emergence of the novel coronavirus SARS-CoV-2 has led to significant interest in its potential transmission between animals and humans, especially pets. This review article summarises the literature on coronavirus infections in domestic animals, emphasising epidemiology, transmission dynamics, clinical manifestations, and public health implications. This article highlights current understandings of the relationship between infections in companion animals and humans, identifies research gaps, and suggests directions for future research. Cases of disease in cats, dogs, and other domestic animals, often occurring through close contact with infected owners, are reviewed, raising concerns about possible zoonotic and reverse zoonotic transmission. Precautions and recommendations for pet owners and healthcare workers are also discussed. The scientific evidence presented in the article highlights the need for a One Health approach that considers the health of people, animals, and the environment to combat future pandemics.

## 1. Introduction

The COVID-19 pandemic, caused by severe acute respiratory syndrome coronavirus 2 (SARS-CoV-2), originated in Wuhan, China, in late 2019 and rapidly grew into a global health crisis. Declared a pandemic by the World Health Organisation (WHO) in March 2020, COVID-19 has resulted in widespread disease, with millions of confirmed cases and a significant number of deaths worldwide [1]. According to the latest data provided by the WHO, as of 10 March 2023, there were approximately 676 million confirmed human cases worldwide, with more than 6.9 million deaths [2]. The disease presents with a spectrum of symptoms ranging from mild, such as fever and cough, to severe, including pneumonia, acute respiratory distress syndrome (ARDS), and death. The pandemic has placed enormous strain on healthcare systems, devastated economies, and led to unprecedented public health measures to control the spread of the virus, including quarantines, travel restrictions, and vaccine development and deployment [3].

The nature of this novel zoonotic virus, its widespread distribution, and the susceptibility of certain animal species to infection are evident in animal infections resulting from close contact between humans and animals. Conversely, there is also evidence that, for some animal species, close contact with infected animals may represent a potential source of infection in humans [2]. The realisation that SARS-CoV-2 can also infect animals, including pets such as cats and dogs, has further complicated the task of understanding and managing the pandemic. Reports of pets becoming infected with COVID-19, mainly from their infected owners, have raised concerns about zoonotic interactions—the two-way transmission of the virus between humans and animals [4,5].

Research into how SARS-CoV-2 spreads between humans and pets and among animals is critical in developing comprehensive strategies to mitigate transmission [6]. Although pets infected with SARS-CoV-2 often exhibit mild or no symptoms, studying these clinical manifestations is vital for veterinary care [7].

Research into the role of pets in COVID-19 transmission is critical to public health. Understanding whether and how pets can contribute to the spread of the virus to humans is essential for developing guidelines that protect the health of both humans and animals and prevent potential cases of reverse zoonosis [8]. The COVID-19 pandemic highlights the need for a One Health approach that considers the interconnected health of people, animals, and the environment. Collaboration between veterinary medicine, human medicine, and environmental sciences is essential to address the complex challenges of pandemics and improve global responses to zoonotic diseases [9].

### 1.1. Transmission of SARS-CoV-2 in Pets

The transmission of SARS-CoV-2 to domestic animals, especially cats and dogs, has been documented, indicating that these animals can acquire the virus from humans. Although cases of pets testing positive for SARS-CoV-2 have been reported worldwide [2], there is limited evidence to suggest the significant transmission of the virus from pets to humans [10]. The primary mode of transmission of COVID-19 remains person-to-person [11].

Susceptibility to SARS-CoV-2 varies significantly among animal species. Research shows that cats, ferrets, and some species of deer are highly susceptible and potentially act as virus carriers. Dogs show lower susceptibility and a reduced ability to transmit the virus [4]. Livestock such as pigs, chickens, and ducks appear to have little susceptibility [11]. These results highlight the importance of monitoring and studying different animal species to understand their role in the virus’s ecology and potential transmission chains.

Evidence of the intra- and inter-species transmission of SARS-CoV-2 has been observed, particularly in mink, where farm outbreaks led to virus mutations that were subsequently transmitted back to humans. This phenomenon highlighted that animals in close contact with humans can serve as reservoirs or mediators for viral mutations [12]. These results also highlight the urgent need for a One Health approach that includes surveillance of animal populations along with monitoring of humans to monitor the spread and evolution of the virus [12].

Factors influencing the transmission dynamics of SARS-CoV-2 in pets include the type of pet (cats and ferrets are more susceptible than dogs), the intensity and type of human–pet interaction (close contact increases risk), the environment in which pets are kept (indoors or outdoors), and the health status of both pets and the people caring for them [13]. These factors contribute to varying levels of risk for pets becoming infected and potentially transmitting the virus within their species or back to humans.

### 1.2. Clinical Signs in Pets

As discussed above, pets, including dogs and cats, can be infected with SARS-CoV-2, which causes COVID-19 in humans. Although pets infected with SARS-CoV-2 often exhibit mild symptoms, some show no symptoms, and it is essential to be aware of common signs that may indicate infection [11]. A study by Shi J et al. showed that, in ferrets, replication was observed in the upper respiratory tract but not in other organs. Cats demonstrated efficient replication and could transmit the virus through airborne transmission, with young cats being more vulnerable than older cats. In dogs, some presence of viral RNA was observed, but there was no evidence of severe infection or effective transmission [4].

The clinical presentation of SARS-CoV-2 infection can vary significantly between animal species, reflecting differences in susceptibility, disease progression, and symptom expression. This variability is influenced by several factors, including the species-specific expression of angiotensin-converting enzyme 2 (ACE2) receptors (which the virus uses to enter cells), differences in the immune system, and variations in viral tropism.

For example, cats and ferrets are more susceptible to SARS-CoV-2 among pets and may exhibit symptoms ranging from mild respiratory problems to gastrointestinal symptoms. Reported symptoms in cats include coughing, difficulty breathing, and lethargy. Ferrets exhibit similar respiratory symptoms; both species can transmit the virus to conspecifics. Dogs appear to be less susceptible to SARS-CoV-2 compared to cats and ferrets. Infected dogs may be asymptomatic or exhibit mild symptoms such as coughing, sneezing, and, in some cases, fever or lethargy [6].

In the wild, some bats are natural hosts for various coronaviruses. However, data on their susceptibility to SARS-CoV-2 are limited. Bats’ unique immune system may allow them to carry viruses without showing clinical symptoms. There have been reports of big cats (such as tigers and lions) testing positive for SARS-CoV-2 in captivity [14,15]. Also, studies and surveillance have identified antibodies to SARS-CoV-2 in free-living deer, suggesting infection and potential asymptomatic spread among these populations [16].

The variability in clinical manifestations across species highlights the importance of ongoing research to understand the dynamics of SARS-CoV-2 transmission and infection in animals. This information is critical for managing public health and ensuring the welfare of domestic and wild animals.

The severity of disease and potential complications of SARS-CoV-2 infection can vary widely among animals, similar to what is observed in humans. The outcome and severity of the disease in pets and other animals depends on various factors, including species, age, underlying health conditions, and possibly breed or genetic predisposition. Although many animals infected with SARS-CoV-2 show mild or no symptoms, there is a risk of more severe effects or complications, especially in some species or individuals with pre-existing conditions.

The same cats and ferrets may exhibit mild to moderate symptoms and can transmit the virus to other animals of the same species. Severe illness is less common, but cats and ferrets with pre-existing health problems may be at higher risk of complications, including pneumonia or severe respiratory distress [4]. Also, older dogs or dogs with underlying health conditions may have a slightly higher risk of developing more severe disease. Among livestock, outbreaks with high mortality rates occurred in mink [17].

Research is ongoing to better understand the impact of SARS-CoV-2 on different animal species, the risks of transmission, and how to best protect animal and human health.

Differences in susceptibility to SARS-CoV-2 infection in animals may be explained by affinity for the ACE2 receptor, which is critical for SARS-CoV-2 entry into host cells. Variations in ACE2 receptor structures among species significantly influence virus binding affinity. For example, ferrets and cats have high-binding-affinity ACE2 receptors, just like humans, making them more susceptible. In contrast, rodents such as mice have lower affinity unless genetically modified to express human ACE2 [18,19]. Variants of the ACE2 gene may also influence susceptibility to and the severity of infection. Specific polymorphisms, such as those found in promoter regions or coding sequences, can alter ACE2 expression levels and function, influencing susceptibility to SARS-CoV-2 in the host organism [20].

Also, differences in susceptibility to and symptoms of infection are influenced by the structure of the respiratory tract, such as the length and pattern of branching, which can affect the distribution of viral load and the manifestation of symptoms. For example, the respiratory anatomy of ferrets is very similar to humans, making them suitable models for studying respiratory viruses [18].

Animals that live close to people, such as pets and zoo animals, are at higher risk of infection. The environment in which animals are kept, whether in captivity or the wild, also influences their exposure and susceptibility [19]. Additionally, animals in the wild experience different environmental stressors and immune problems compared to animals in captivity, which may influence their susceptibility and severity of symptoms. Differences in susceptibility to SARS-CoV-2 infection and symptom expression among animals are multifaceted and include genetic predisposition, anatomical and physiological differences, differences in immune responses, and environmental factors.

### 1.3. Epidemiology of COVID-19 in Pets

**Figure 1:**
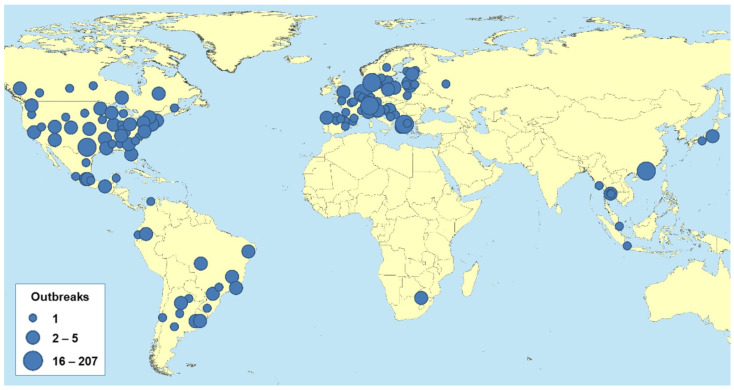
The distribution of SARS-CoV-2 outbreaks worldwide has been reported to WOAH (as of 30 June 2023). The size of the points on the map is proportional to the number of registered flares [2].

Comprehensive data on the global prevalence and incidence of SARS-CoV-2 infections in domestic animals have been limited and vary widely across regions and over time. This is because differences in surveillance efforts, the limited availability of animal testing, inconsistent reporting standards, and fluctuating incidence contribute to the limited and inconsistent data. Figure 1 shows the geographic distribution of SARS-CoV-2 outbreaks in animals reported to WOAH. Hong Kong (SARC) officially reported the first case of SARS-CoV-2 in animals to WOAH on 29 February 2021, in a dog.

Worldwide, 775 animal outbreaks have been reported, affecting 29 species in 36 countries (Table 1). It is important to emphasise that disease detection in animals depends mainly on the level of surveillance at the country level; therefore, these figures are likely to be underestimated. Some countries have seen a high prevalence of outbreaks in mink farms, and variant strains have now been identified in martens. Because SARS-CoV-2 infection is an emerging disease, WOAH strongly encourages members to report, through WAHIS, the occurrence of any cases in animals that meet the case definition in the WOAH guidelines [2].

Monitoring and reporting SARS-CoV-2 infections in animals, including companion animals such as cats and dogs, is less systematic than in humans, making global assessments difficult. However, cases of SARS-CoV-2 infection in pets have been confirmed in several countries, including but not limited to the United States, Canada, Brazil, European countries, and parts of Asia.

These cases have typically been identified in pets living in households with confirmed human cases of COVID-19, which, in most cases, involves human-to-animal transmission. The first evidence of the natural infection of dogs with SARS-CoV-2 was reported in Hong Kong, confirmed by viral RNA [6].

Serological studies testing the presence of antibodies to SARS-CoV-2 have shown varying prevalence rates among pets in different regions. These studies suggest that many pets may have been exposed to the virus, especially those living in households with people infected with COVID-19. Prevalence rates in pets vary widely among published studies, reflecting differences in sample sizes, geographic regions, and periods.

Four outbreaks were reported in three countries (Argentina, Ecuador, Italy) in four animal species (American mink, great hairy armadillo, black-headed spider monkey, and common woolly monkey) during the latest WOAH reporting period (1 April 2023–30 June 2023) [2].

### 1.4. Summary of SARS-CoV-2 Infections in Companion Animals and Their Implications

In summary, although data on SARS-CoV-2 infections in companion animals are available worldwide, comprehensive global data on prevalence and incidence rates are limited due to various factors, including differences in surveillance efforts, testing availability, and reporting standards. Continued monitoring and research are needed to understand the impact of the virus on animal health and its implications for public health.

To some extent, the geographic distribution and temporal trends of SARS-CoV-2 infections in pets reflect the dynamics of the pandemic in humans. However, detailed and complete data specifically for companion animals are more limited and may vary by region, reflecting differences in testing availability, public awareness, and reporting protocols.

Cases of SARS-CoV-2 infection in pets have been reported worldwide, including in North America, Europe, Asia, and South America. These infections were mainly found in domestic animals such as cats and dogs.

The first reports of pets (especially cats and dogs) testing positive for SARS-CoV-2 appeared in early 2020, shortly after the pandemic began to impact the global population. As the pandemic progressed, reports of pet infections increased, especially in regions with a surge in human cases.

Temporal trends in infections in companion animals are closely related to trends in human diseases. Increases in the number of cases in pets have often been observed following a sharp rise in the number of cases in humans, suggesting that human-to-pet transmission is the main route of infection.

Over time, increased surveillance, research, and public awareness have led to the better documentation and understanding of SARS-CoV-2 infections in companion animals. Various studies have aimed to assess the prevalence of antibodies against SARS-CoV-2 in pets and have shown that many pets in households affected by COVID-19 may have been exposed to the virus.

A problem and limitation of research into the virus’s effects on pets is underreporting. The number of SARS-CoV-2 infections in pets is likely underestimated due to limited testing and the presence of asymptomatic or mild cases that may not be recognised by pet owners or reported to authorities.

Thus, although there is a global distribution of SARS-CoV-2 infections in companion animals, detailed data on geographic distribution and temporal trends are influenced by factors such as local human infection rates, testing availability, and surveillance efforts. The relationship between trends in the spread of COVID-19 in humans and infections in pets highlights the importance of comprehensive surveillance and research to understand and manage the health impacts of the pandemic on both humans and animals.

**Figure 2:**
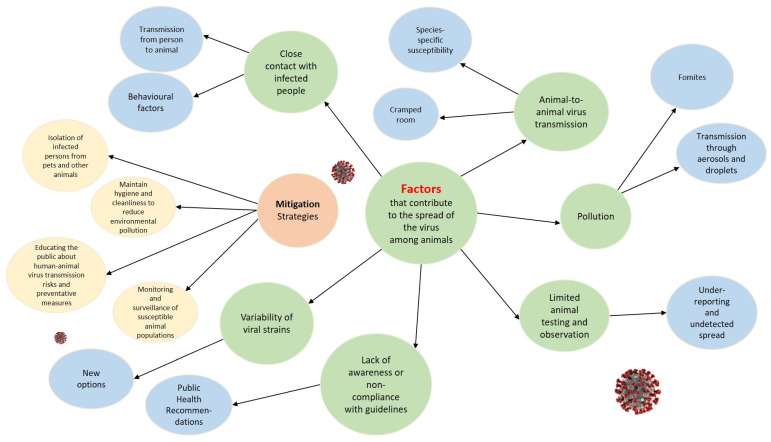
Flowchart representing the factors contributing to the spread of the virus among animals. The flowchart visually organises the primary factors and their sub-points, providing a clear overview of the interconnected nature of animal virus transmission.

The spread of SARS-CoV-2 among animals, especially pets and other companion animals, is influenced by several factors that facilitate transmission. Understanding these factors is critical to developing strategies to minimise interspecies transmission and protect animal and human health (Figure 2).

The above approaches and strategies developed in the context of COVID-19 also have broad applications in the management of other zoonotic diseases affecting both domestic and wild animals. Using the example of the COVID-19 pandemic, one can clearly see how important interdisciplinary interaction and collaboration are within the framework of the One Health concept. This approach not only strengthens public health systems, but also contributes to the better understanding and control of zoonotic infections in general.

Factors such as close contact between humans and animals, the ability of pathogens to transmit between species, and the dynamics of infection in different environments are key in addressing any zoonotic threats. Understanding these aspects is critical to developing effective prevention and response strategies that take into account not only human health, but also animal health and the environment.

The implementation of strategies such as improved animal health monitoring, the development of veterinary diagnostics, educational programs to increase public awareness of zoonoses, and the increased regulation and control of animal welfare conditions can significantly reduce the risk of zoonotic infections. These measures, which have been successfully used in the fight against COVID-19, should become part of a standard One Health approach to health management, applicable to a wide range of zoonotic pathogens affecting animals and humans.

### 1.5. Public Health Implications

The zoonotic potential of SARS-CoV-2, particularly concerning transmission from domestic animals to humans, has been the subject of significant interest and research since the onset of the COVID-19 pandemic. Based on current understanding and evidence, the risk of transmission of SARS-CoV-2 from pets to humans is considered low. However, the dynamic nature of virus evolution and transmission means that ongoing research and surveillance are necessary to completely understand and manage these risks.

Pets play a unique role in households, influencing disease transmission dynamics, including SARS-CoV-2, the virus responsible for COVID-19. Although the primary mode of transmission of COVID-19 is person-to-person, the presence and behaviour of pets in the home can directly and indirectly affect transmission dynamics.

Ideas regarding the role of domestic animals in the dynamics of transmission in the home include the following:

(1) Direct interaction and transmission

Transmission from humans to domestic animals. Cases of pets, especially cats and dogs, becoming infected with SARS-CoV-2 through close contact with infected people have been reported. This scenario highlights the potential for domestic animals to participate in the domestic transmission cycle, primarily as recipients of the virus from humans.

Transmission from pet to pet. Once a pet is infected, the virus can potentially spread to other pets in the same household, especially to species known to be susceptible to SARS-CoV-2, such as cats and ferrets. This intra-household transmission among animals could potentially prolong the duration of the virus’s presence in the home.

Pet-to-human transmission: Although the risk is considered low, there are concerns about the possibility of virus transmission from pets to humans. However, according to the latest data available, there is limited evidence to suggest significant transmission from domestic animals to humans. Continued research is needed to understand this risk fully.

(2) Indirect influence on the dynamics of transmission

Behavioural practices. The way people interact with their pets can influence disease transmission dynamics. For example, people infected with SARS-CoV-2 are advised to limit close contact with their pets, similar to recommendations for interactions between people, to reduce the risk of transmission.

Role in human social interactions: Pets often play a central role in social interactions within and between households. Dog walking can increase social contact between people from different families, potentially influencing broader SARS-CoV-2 transmission dynamics.

Emotional and psychological support. Pets provide significant emotional and psychological support, especially during social isolation and stress, such as quarantine. This support can influence people’s behaviour and compliance with public health measures, indirectly influencing transmission dynamics.

Although pets may play a role in the transmission dynamics of SARS-CoV-2 in the home, their influence is influenced by human behaviour, the species of pets, and the nature of human–pet interactions. Following public health recommendations can help minimise the risk of transmission from pets. Continued research and surveillance are critical in fully understanding these dynamics and ensuring people’s, and their pets’, health and well-being.

The One Health approach is a collaborative, multisectoral, and transdisciplinary strategy that works at local, regional, national, and global levels to achieve optimal health outcomes by recognising the interconnectedness between people, animals, plants, and their shared environments. In combating the COVID-19 pandemic, particularly regarding companion animals, the One Health approach emphasises the importance of integrating animal health, human health, and environmental health practices to prevent and control the spread of SARS-CoV-2 and fully understand its consequences.

### 1.6. Management and Prevention Strategies

The treatment of COVID-19 in pets, especially cats and dogs, where SARS-CoV-2 infection from humans has been confirmed in some cases, primarily focuses on supportive care, because most infected pets have mild symptoms and some are asymptomatic. According to the latest data, no specific antiviral drugs are approved for the treatment of COVID-19 in animals. Treating COVID-19 in pets relieves symptoms and prevents secondary infections, ensuring the animal’s comfort and full recovery.

To prevent the virus from spreading to other pets and people, it is recommended that the infected pet is isolated from other animals and people, except its caregiver, during the infectious period. Although antibiotics do not treat viral infections, they may be prescribed by a veterinarian if a secondary bacterial infection is suspected or confirmed. Good hygiene practices, including washing hands before and after handling a pet, cleaning and disinfecting the pet’s area, and using personal protective equipment (PPE) when recommended, can help prevent the spread of the virus.

The widespread vaccination of pets against SARS-CoV-2 has not been standard practice worldwide. The main focus of vaccination against COVID-19 has been on people, given the significant impact of the virus on human health and the dynamics of primary transmission of the disease. However, the veterinary and scientific communities continue to develop and discuss strategies for vaccinating animals, especially those at higher risk of infection or that may act as reservoirs for the virus. The targeted vaccination of high-risk groups can be carried out. For example, minks, which have been involved in significant outbreaks of SARS-CoV-2 and could potentially transmit it back to humans, have become the target of vaccination campaigns in some countries. Also, some animals in zoos or research facilities, especially those known to be susceptible to SARS-CoV-2 (such as apes and felids), may be vaccinated as a precaution.

Research has been conducted to develop vaccines specifically for animals [51]. For example, Russia has registered the first COVID-19 vaccine for animals, called Carnivac-Cov, designed to protect species such as cats, dogs, and minks [52]. Also, Kazakh scientists have developed and assessed the safety of the NARUVAX-C19 (pets) vaccine, which is a vaccine that is based on the extracellular domain of a recombinant spike protein expressed in insect cells and then formulated with appropriate adjuvants [53]. Other countries and companies are also studying or developing animal vaccines.

Any vaccine developed for animals must undergo rigorous testing to ensure its safety and effectiveness. This process involves assessing the vaccine’s ability to elicit an immune response in the animal without causing serious side effects.

Vaccination strategies prioritise human vaccines to control the population pandemic. Pet vaccines must be considered in the broader context of public health priorities and virus dynamics.

### 1.7. Future Research Directions and Needs

The COVID-19 pandemic has highlighted the need for comprehensive research to better understand the virus’s impact on pets and how to control it effectively. Addressing this problem requires a multifaceted research approach that includes developing new diagnostic tools, therapeutic interventions, long-term monitoring, and collaboration between veterinary and public health authorities.

By addressing these areas through targeted research and collaboration, we can improve our understanding of COVID-19 in companion animals, improve diagnostic and treatment options, and develop effective strategies to mitigate the virus’s impact on companion animal populations and human health. This comprehensive approach will also strengthen our preparedness for future pandemics associated with zoonotic diseases.

## 2. Material and Methods

### 2.1. Literature Search

A systematic search of scientific databases like PubMed, Scopus, and Web of Science was conducted using keywords such as “COVID-19 in pets”, “SARS-CoV-2 transmission animals”, and “zoonotic transmission”. Relevant articles from 2019 to 2023 were selected for review, including studies on SARS-CoV-2 infections in wild and domestic animals, epidemiology, transmission dynamics, clinical manifestations, and the public health implications of COVID-19 in pets.

### 2.2. Species Susceptibility

We reviewed studies on the susceptibility of various animal species to SARS-CoV-2, including experimental infection and observational studies on cats, dogs, ferrets, and minks, as well as data on virus replication and transmission efficiency in different species.

### 2.3. One Health Framework

We emphasised the importance of a One Health approach, integrating human, animal, and environmental health, and highlighted collaborative efforts between veterinary and public health authorities to develop comprehensive strategies for managing the pandemic and preventing future zoonotic outbreaks.

### 2.4. Data Extraction

Data from the selected studies were categorised into sections: epidemiology, trans-mission dynamics, clinical manifestations, and public health implications. Key findings, study designs, sample sizes, and outcomes were recorded.

## 3. Conclusions

This review highlights the importance of the One Health approach in the COVID-19 pandemic, highlighting the complex relationships between human, pet, wildlife, and environmental health. Although current evidence indicates a relatively low risk of transmission of SARS-CoV-2 from pets to humans, further research is needed to fully understand the virus and its impact on different animal species.

In addition, the study highlights the importance of monitoring, diagnosing, and, if necessary, developing vaccines to protect domestic and wild animals from the virus. This could help prevent future outbreaks and protect both animal and human populations. This study also highlights the need for interdisciplinary and cross-sector collaboration to develop and implement effective public health strategies considering animal health and its associated risks for humans.

In conclusion, the COVID-19 pandemic demonstrates that human health is inextricably linked with animal health and the health of the environment. The One Health approach offers a framework for the collaboration and integration of human, veterinary, and environmental health efforts to build resilient health systems that can effectively respond to future zoonotic and pandemic threats.

## Figures and Tables

**Table 1:** Number of outbreaks (n = 775) reported worldwide, by species and region (as of 30 June 2023).

	Regions	Africa	America	Asia	Europe	References
Animal						
Binturong (Catbear)			X			[21]
Large hairy armadillo			X			[22,23,24]
Black-headed spider monkey			X			[25,26]
Black-tailed marmoset			X			[27,28]
Lynx			X			[29,30,31]
Cat			X	X	X	[4,32,33]
Dog			X	X	X	[4,33]
Fishing cat			X			[21]
Giant anteater			X			[34]
Gorilla			X		X	[35,36]
Hamster				X		[37]
Hippopotamus					X	[38,39]
Lion		X	X	X	X	[40,41,42]
Mink			X		X	[12,43]
Mule deer			X			[36]
Otter			X			[21]
Domestic ferret			X		X	[4]
Puma		X	X			[41]
Red fox					X	[23,44]
Snow leopard			X			[45]
South American coati			X			[21,46]
Tiger			X	X	X	[47,48]
West Indian manatee			X			[49]
White-tailed deer			X			[50]
